# Silicone Rubber Composites Reinforced by Carbon Nanofillers and Their Hybrids for Various Applications: A Review

**DOI:** 10.3390/polym13142322

**Published:** 2021-07-15

**Authors:** Vineet Kumar, Md Najib Alam, Amutheesan Manikkavel, Minseok Song, Dong-Joo Lee, Sang-Shin Park

**Affiliations:** 1 School of Mechanical Engineering, Yeungnam University, 280, Daehak-ro, Gyeongsan 38541, Korea; vineetfri@gmail.com (V.K.); mdnajib.alam3@gmail.com (M.N.A.); mamutheesanbh@gmail.com (A.M.); djlee@yu.ac.kr (D.-J.L.); 2 Graduate School of Mechanical Engineering, Yeungnam University, 280, Daehak-ro, Gyeongsan 38541, Korea; masonsong616@gmail.com

**Keywords:** carbon nanotubes, graphene, carbon black, silicone rubber, nanocomposite properties, strain sensors

## Abstract

Without fillers, rubber types such as silicone rubber exhibit poor mechanical, thermal, and electrical properties. Carbon black (CB) is traditionally used as a filler in the rubber matrix to improve its properties, but a high content (nearly 60 per hundred parts of rubber (phr)) is required. However, this high content of CB often alters the viscoelastic properties of the rubber composite. Thus, nowadays, nanofillers such as graphene (GE) and carbon nanotubes (CNTs) are used, which provide significant improvements to the properties of composites at as low as 2–3 phr. Nanofillers are classified as those fillers consisting of at least one dimension below 100 nanometers (nm). In the present review paper, nanofillers based on carbon nanomaterials such as GE, CNT, and CB are explored in terms of how they improve the properties of rubber composites. These nanofillers can significantly improve the properties of silicone rubber (SR) nanocomposites and have been useful for a wide range of applications, such as strain sensing. Therefore, carbon-nanofiller-reinforced SRs are reviewed here, along with advancements in this research area. The microstructures, defect densities, and crystal structures of different carbon nanofillers for SR nanocomposites are characterized, and their processing and dispersion are described. The dispersion of the rubber composites was reported through atomic force microscopy (AFM), transmission electron microscopy (TEM), and scanning electron microscopy (SEM). The effect of these nanofillers on the mechanical (compressive modulus, tensile strength, fracture strain, Young’s modulus, glass transition), thermal (thermal conductivity), and electrical properties (electrical conductivity) of SR nanocomposites is also discussed. Finally, the application of the improved SR nanocomposites as strain sensors according to their filler structure and concentration is discussed. This detailed review clearly shows the dependency of SR nanocomposite properties on the characteristics of the carbon nanofillers.

## 1. Introduction

Next-generation carbon-based nanofillers have gained great importance owing to their excellent properties that are suited to a wide range of applications, such as strain sensing. Yang et al. [1] showed the improved strain sensing behavior of composites based on graphene (GE)-filled silicone rubber (SR). Moreover, Kumar et al. [2] showed that the use of carbon nanotubes (CNTs) as a nanofiller in an SR matrix leads to improved mechanical and electrical properties. Furthermore, Kumar et al. [3] demonstrated a piezoresistive strain sensor based on CNTs and carbon black (CB) in an SR matrix, and the improved gauge factor and durability of the composites were studied. Lastly, Song et al. [4] demonstrated the strain sensing behavior of CNT/CB hybrid in an SR matrix, and the high strain sensitivity of the composites was demonstrated. In recent years, strain sensors have been extensively investigated due to their promising applications in health monitoring [5], flexible electronic devices [6], or detecting locomotive motions [7]. Recently, flexible strain sensors based on polymer or rubber composites have been favorable for the above applications. These strain sensors based on polymer composites offer various advantages, such as high flexibility [8], robust stretchability [9], high gauge factor [3], and high sensitivity [10]. Promising strain sensors probably exhibit a high sensitivity to compressive or tensile strain originating from various mechanical motions [7].

**Scheme 1:**
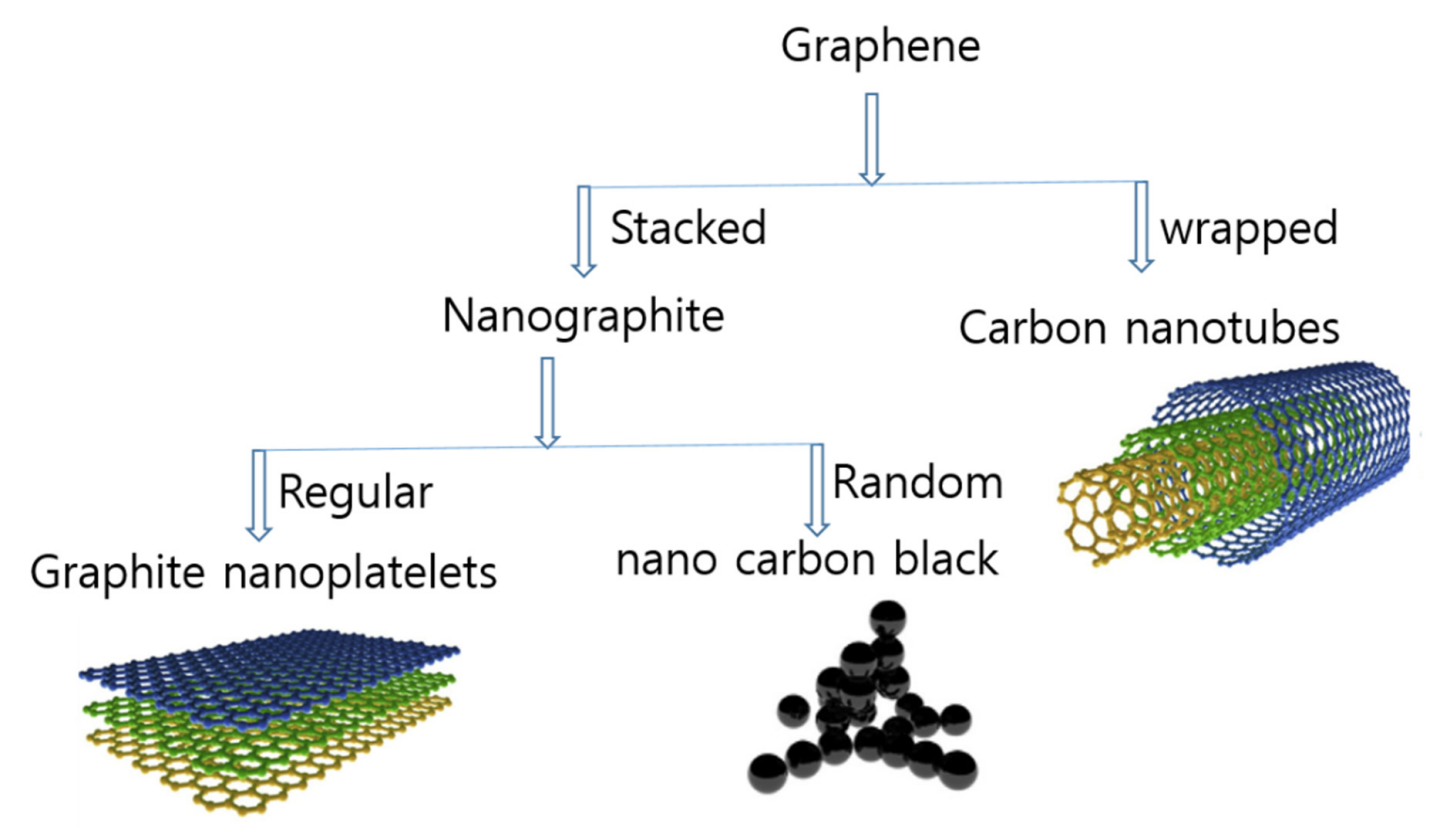
Graphene, the mother of all carbon nanomaterials.

Polymer composites are based on various types of polymers, which are typically elastomers, such as SR, while nanofillers, such as GE [10] or their hybrid [11] and CNTs [12], are added to improve the conductivity of the composites. In particular, much attention has been paid to carbon nanotubes (CNTs), namely by Zhou et al. [13], Earp et al. [14], Wei et al. [15], and Song et al. [16]. All these authors demonstrated the successful use of CNTs as a nanofiller, and improved properties were studied. Similarly, the use of GE as a nanofiller was shown by Ren et al. [17], Liao et al. [18], Zhang et al. [19], and Wei et al. [20], and improved properties were reported that are amenable to the development of next-generation polymer composite materials. The small particle size [21], high aspect ratio [22], and optimum surface area [23] of these nanofillers allow for the achievement of good mechanical and electrical properties at low filler content, as demonstrated by Khanna et al. [24], Wang et al. [25], Wu et al. [26], and Wei et al. [27]. These authors reported CNTs as a single or hybrid filler in various matrices, and improved properties were reported. Thus, to produce innovative material systems with excellent properties, these nanofillers were used as reinforcing agents in the rubber matrix. Graphene has emerged as the mother of all carbon allotropes [28]. When stacked, few-layer or multilayer graphene (MLG) is formed [29]; when randomly stacked, different grades of carbon black (CB) are formed [30]; and when wrapped, single- or multilayer carbon nanotubes result (Scheme 1). However, the nanofillers tend to aggregate at high contents owing to intermolecular Van der Waals interactions [31] among the single or hybrid filler particles [32], resulting in poorer properties [33]. Thus, the nanofiller must be optimized in order to obtain the best mechanical and electrical properties.

SR is composed of a base and a vulcanizing agent with variable hardness [34]. The most promising feature of SR is its low viscosity, ease of processing, high heat and acid aging resistance, and good flexibility [35,36]. Thus, SR is traditionally used as a host matrix in nanocomposites reinforced with carbon nanomaterials for various applications, such as strain sensing [1,2]. With their high aspect ratio, high filler networking density, and favorable morphology, CNTs have emerged as the best candidate among the different carbon nanofillers for SR and are promising for different applications [36,37]. The beneficial effect of these nanofillers on the mechanical properties of SR makes them a promising alternative to traditional fillers [38] such as CB [39,40]. The improved mechanical properties include the compressive modulus, tensile strength, reinforcing factor, experimental deviation of the Young’s modulus from the theoretical modeling, filler percolation threshold, heat dissipation losses, and stress transfer from SR polymer chains to filler particles. Although the mechanism of reinforcement is not fully understood, the basic principles governing the stress–strain behavior in filled nanocomposites are known. In addition to the improved modulus of the filled rubber with the inclusion of filler in soft SR [41], the interfacial interactions and filler–filler interactions [42] in the nanocomposite also contribute to the reinforcement [43].

At a low range, the nanocomposite microstructure can be studied through scanning electron microscopy (SEM), transmission electron microscopy (TEM), and atomic force microscopy (AFM). At the medium range, storage is evaluated using the Rubber Process Analyzer (RPA), and the storage modulus, loss modulus, and tan δ are calculated, while long-range investigations include stress–strain curves. The behavior at a large strain is described by the strain amplification effects. At a lower range (<1% strain), the filler–filler interactions and elastic range of nanocomposites can be observed in the stress–strain curve or through the Payne effect, as shown by Narayan et al. [44], Jalocha et al. [45], and Xu et al. [46]. These authors theoretically and experimentally demonstrated that at higher strains (>1 to 100%), interfacial interaction is assumed in the Payne effect, and at higher strain (>100% strain), the effect of the in-rubber structure on the mechanical properties is demonstrated. The physical performance of rubber nanocomposites strongly depends on the filler morphology [47,48], interfacial interaction, and surface area, as well as the polymer–filler compatibility [49,50]. However, the most important factor is the interfacial interaction, which enables stress transfer from the polymer chains of SR into filler particles such as GE or CNT. These polymer–filler interactions, which are based on the adsorption of polymer chains on the surface of filler particles, can be controlled by the interfacial area. The high surface area of the filler provides a more favorable surface for the filler to interact with the polymer chains [49,51].

Although numerous investigations have been devoted to carbon nanofillers, SR matrices [52], nanocomposites based on them, and the use of these nanocomposites in various applications have dominated the discussion [53,54]. Generally, CB is used as a reinforcing filler to improve the properties of rubber composites, but a high content is required for obtaining optimal properties. This high content alters the properties of rubber composites, such as stress relaxation. Thus, in this paper, nanofillers that provide optimal properties at as low as 2–3 phr are reported and reviewed. This review attempts to bridge the studies on the use of these nanofillers reported in the literature by presenting the results for the best fillers or nanocomposite systems. The advantages of using nanofillers and choosing SR as the rubber matrix for applications such as strain sensors are discussed. The detailed mechanical, thermal, tribology, and electrical properties of SR filled with different carbon nanofillers under static and cyclic strains, along with their suitability for strain sensor applications, are also reviewed. Through this approach, CNT-based composites were found to exhibit the best mechanical and electrical properties and are, thus, the most suitable for high-performance applications.

The potential advantages of using SR reinforced with carbon nanomaterials used in industrial applications such as strain sensors are as follows:

- The shore A hardness of virgin SR and, in some cases, filled SR is below 65. Therefore, it can be used in various soft applications, such as actuators or strain sensors, etc. [55].
- Virgin SR is easy to process, soft, has good flexibility and good tensile strength, and is easy to vulcanize. Therefore, it is promising to be used as a strain sensor [56].
- SR has high thermal stability, good aging properties, and low environmental degradability [57].
- Virgin SR is an insulator. Therefore, although it has advantages for use in electric cables, it cannot be used as a strain sensor. As such, conducting nanofillers are added to make the rubber-matrix-based composite electrically conductive to make it useful for strain sensors [58,59].
- Virgin SR is ductile. Therefore, it is vulcanized to be useful for various industrial applications, such as strain sensors. However, the type of vulcanization, such as room-temperature-vulcanized silicone rubber [36] and high-temperature-vulcanized silicone rubber [36], not only affects the mechanical properties, such as the mechanical stiffness of the rubber composites, but also affects the strain sensing properties, such as the gauge factor, stress relaxation, and durability [3].
- The main disadvantage of using SR is its poor fracture strain. Most often, the fracture strain of silicone rubber is up to ~300%, which is too short as compared to that of natural rubber [60]. Another disadvantage of using silicone rubber is the dependency of the mechanical properties of silicone rubber on the type of vulcanization [36]. Various advantages of using silicone rubber are described in the above section.

## 2. Characterization of Carbon Nanofillers

### 2.1. Microstructure, Defect Density, and Crystalline State of the Carbon-Based Filler Particles

**Figure 1:**
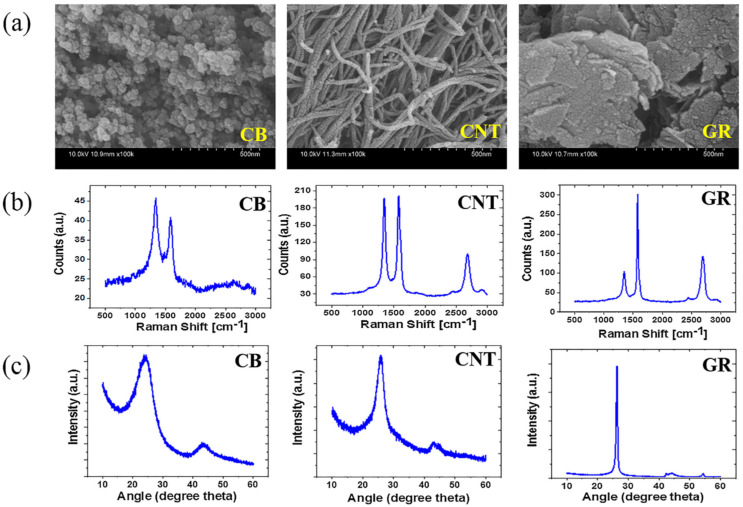
(**a**) SEM micrographs, (**b**) Raman spectra, and (**c**) XRD patterns of CB, CNT, and GR [3].

CB is roughly oval and predominantly consists of spherical particles with different diameters and particle sizes, as shown in Figure 1a. The spherical CB particles are distributed unevenly, and their aggregation has been observed [61]. The spherical particles become larger as the number of particles in the aggregates increases [62]. CB exhibits structural lengths varying from a few angstroms to micrometers [3]. CB is a traditional filler material used in a wide range of polymer matrices, including SR. CB optimally reinforces the SR matrix, but a high amount is required, which leads to undesirable composite properties such as high hardness, processing difficulty, and high viscosity [63]. Therefore, nano carbon black (nano-CB) has recently been used by various researchers to obtain favorable properties at low loadings [2].

CNTs can be formed by wrapping graphene or multilayer graphene to form single-walled CNTs [64] or multi-walled CNTs (MWCNTs) [65], respectively. CNTs typically have a diameter below 20 nm and lengths ranging from a few nanometers to micrometers; this yields a high aspect ratio of approximately 65 [2,3]. These features make CNTs ideal nanofillers for reinforcing the SR matrix, and the properties improve exponentially as the aspect ratio is increased [66]. The tubes are highly entangled and randomly distributed, as shown in the SEM image in Figure 1a. The length, diameter, and distribution of the tubes can be identical or different depending on the synthetic method. CNTs have attracted great attention as nanofillers for extremely strong, ultra-lightweight, and high-performing SR nanocomposites for various applications, such as strain sensing.

Nanographite (GR) has a sheet-like morphology with different numbers of graphene layers stacked together [67]. These GR sheets have a thickness of a few nanometers (basically below 10 nm) and lateral dimensions from hundreds of nanometers to submicrons, leading to a high aspect ratio of 5–10 [3]. The SEM micrograph of GR shown in Figure 1a reveals that the graphene layers are stacked in an orderly manner, as seen in graphite or expanded graphite. Different terminology is used to refer to GR in the literature, such as monolayer, bilayer, or trilayer GE, and few-layer graphene (FLG) for <5 layers of stacked graphene [68]. Other terms include MLG for <10 stacked layers, graphene nanoplatelets (GNPs), expanded graphite (EG), or graphite nanoflakes (GNFs) for stacked graphene layers with thicknesses below 100 nm [68]. The degree of exfoliation of the graphene layers in GR strictly depends on the method of synthesis [3].

Raman spectroscopy is generally employed to assess the structural properties of GR-based carbon nanomaterials, such as defect density, defect structure, and number of graphene stacked layers [69]. Studies have found that the structural properties depend on the type and nature of the carbon nanomaterial investigated (Figure 1b). A significant change in the defect density and defect structure was reported to take place between the different types of carbon nanomaterials [70]. These structural changes are clearly reflected in the Raman signatures of the CB, CNT, and GR nanomaterials (Figure 1b).

The Raman spectra display three bands (D-band, G-band, and 2D band) at different Raman shifts as characteristic features of CB [71], CNT [72], and GR [73]. The Raman spectra of CNT and GR show two different phonon oscillations in the G-band and 2D band, but for CB, the 2D band is absent because it is extremely sensitive to changes in structural properties based on interlayer interactions. Moreover, the full width at half maximum of the 2D GR signifies the number of graphene layers stacked in the crystalline domain regime. The D-band is directly related to the defect density and structural defects present in the carbon nanomaterials. As indicated in Figure 1b, the D-band and its intensity are directly related to the amount of disorder present in CB, CNT, and GR. The I_D_/I_G_ ratio, which is the intensity ratio of the D/G-bands, was 1.12 for CB, 1.05 for CNT, and 0.3 for GR, indicating that the lowest defect density occurs for GR and the highest for CB-based carbon nanomaterials [3]. It should be noted that the number of defects increases from GR to CB, thereby affecting the properties of CB-based SR composites more than those of CNT- and GR-based composites.

X-ray diffraction (XRD) is generally employed to investigate the crystalline state of carbon nanofillers. A prominent (002) peak usually appears at 2θ = 26°, which is the characteristic peak for carbon nanomaterials (Figure 1c). The dimensions of crystallite GR in the orthogonal and parallel directions with respect to the structural layers can be estimated using XRD [74]. The intensities of the reflections in XRD patterns are generally not correlated to polarization or Lorentz factors in order to allow better visibility of (00ℓ) peaks. The d-spacing can be estimated using Bragg’s law, and the D_hkℓ_ correlation length can be estimated using Scherrer’s equation, as follows:

D_hkℓ_ = Kλ/(β_hkℓ_ cos θ_hkℓ_), (1)

where K is Scherrer’s constant, λ is the wavelength of the irradiating beam (1.5419 Å, CuK_α_), β_hkℓ_ is the width at half height, and θ_hkℓ_ is the diffraction angle. A correction factor must be used if θ_hkℓ_ is lower than 1°. D_00ℓ_ can be obtained by using the (002) reflections. By estimating D_hkℓ_ and assuming a 0.33-nanometer interlayer spacing, the number of graphene layers stacked in GR and wrapped in CNTs can be estimated. The XRD pattern of GR shows highly ordered features, in which 20–25 graphene layers are generally stacked in the crystalline domain region [3]. Similarly, CNTs show multilayer (35–40 layers) graphene stacking wrapped in a concentric form in the crystalline domain of CNTs [3]. However, the number of layers in CB cannot be estimated using Scherrer’s equation because of the random stacking of graphene layers. Moreover, the XRD results reveal that there is an appreciable amount of amorphous carbon in the CB specimens.

### 2.2. FTIR, XPS, and XRD of Graphite, GO, GE

**Figure 2:**
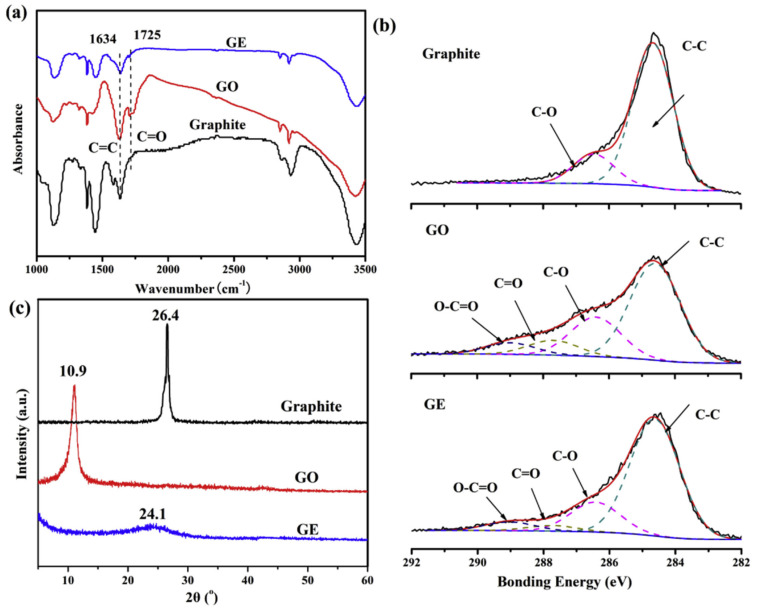
(**a**) FTIR, (**b**) XPS, and (**c**) XRD patterns of graphite, GO, and GE [75].

Monolayer, bilayer, and FLG can be synthesized by reduction of graphene oxide (GO) obtained from bulk graphite. FTIR is a promising technique to investigate the chemical changes that occur during the oxidation of graphite to GO and the reduction into GE (Figure 2a). Compared to graphite, a new absorption peak at 1725 cm^−^^1^ (assigned to C=O) was seen in graphite oxidizing to GO. However, this absorption peak (C=O peak) disappears while reducing GO to GE. It shows that there is reduction in oxygen-containing functional groups in the process of reducing GO to GE. XPS is further used as a tool to classify the quantitative details about the type and functionalization of GO and GE. The XPS survey for graphite, GO, and GE is reported in Figure 2b. The peak with high intensity at ~284.6 eV represents the sp^2^ and sp^3^ hybridized carbon atoms. The other distinguished peaks at ~286.5, ~287.7, and ~289.1 eV typically correspond to C-O, C=O, and O-C=O, respectively [75]. The O/C ratio on the surface of the specimens can be extracted through the ratio of respective peak areas. The extracted O/C ratio for graphite, GO, and GE is 11.2, 35.6, and 18.2%, respectively [75]. The significant increase in ratio for GO is due to the oxidation of graphite, which increases the amount of oxygen-containing functional groups. However, a decrease in the O/C ratio in GE confirms the successful reduction of GO into GE.

Further studies on the successful oxidation of graphite and its reduction into GE were performed using XRD. The XRD patterns of graphite, GO, and GE are presented in Figure 2c. It was found that pristine graphite shows a sharp peak at 26.4°, indicating the ordered structure of graphite. After oxidation of graphite into GO, the peak shifts to 10.9°, with an interlayer distance of 0.85 nm [75]. With the further process of reducing GO into GE, the weak peak at 24.1° of reduced GE highlights the restoration of the graphitic structure with significant exfoliation in its structure [75].

## 3. Processing of SR/Carbon-Nanofiller-Based Nanocomposites

### 3.1. Solution Casting Processing

**Scheme 2:**
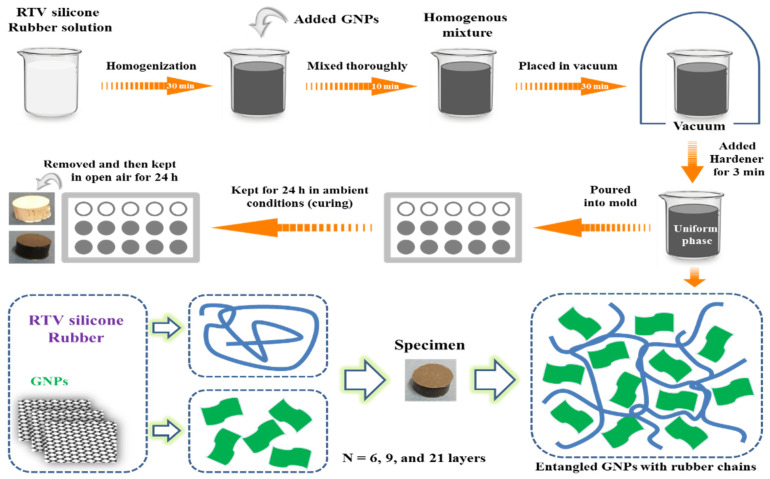
Schematic presentation of the preparation of RTV-SR-based nanocomposites and the suggested mechanism for reinforcement of the RTV-SR matrix with carbon nanofillers.

Nanocomposites are prepared by mixing a solution of room-temperature-vulcanized (RTV)-SR with carbon nanofillers via solution casting. The sample preparation process is initiated by spraying the molds with a mold-releasing agent. The molds are sprayed and dried for 2 h. For the preparation of rubber nanocomposites, carbon nanofillers are mixed with an SR solution for 10 min. The SR/carbon-nanofiller nanocomposites are then subjected to vacuum for 30 min to remove air cavities from the SR matrix. Subsequently, 2 phr of vulcanizing agent is added, and the mixture is poured into the sprayed mold, which is then manually pressed firmly and kept for 24 h at room temperature for vulcanization. Samples are then removed from the sprayed molds and the mechanical and electrical properties are tested, as in our previous study [76,77]. A schematic of the procedure for the preparation of SR-based nanocomposites is shown in Scheme 2.

### 3.2. Dispersion of Carbon Nanoparticles in SR Composites

#### 3.2.1. Filler Dispersion through AFM

**Figure 3:**
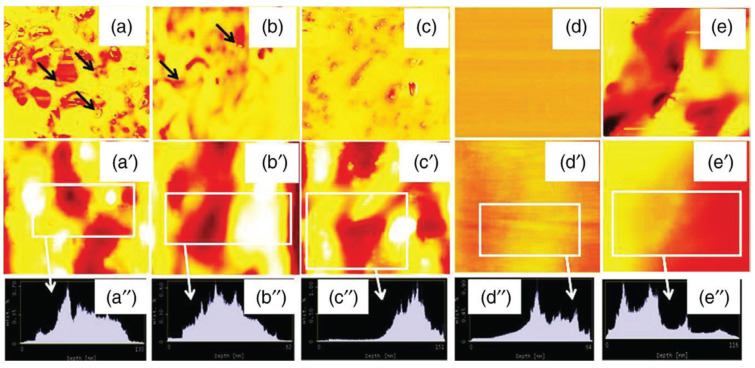
AFM micrographs in RTV-SR matrix: (**a**,**a’**,**a’’**) CB + GR hybrid, (**b**,**b’**,**b’’**) CB + CNT hybrid, (**c**,**c’**,**c’’**) CB, (**d**,**d’**,**d’’**) GR, and (**e**,**e’**,**e’’**) CNT [42].

AFM is an important tool for studying the substrate depth, grain size, and roughness of filled polymer composites. Figure 3 shows the use of AFM to investigate the dispersion of filler particles [78], such as CNTs, GR, CB, and their hybrids. The phase images (Figure 3a–e), topological images (Figure 3a’–e’), and histograms of the grain heights (Figure 3a’’–e’’) are presented. As seen in the phase images, the CNT and GR particles in the hybrid systems were found to be preferentially located near CB filler particles (black arrows, Figure 3a,b). The migration of CNT and GR particles towards CB particles was proposed to form synergistic filler networks, leading to synergistic effects of the mechanical and electrical properties of the composites. The topological images of the composites show the distribution of GR particles in exfoliated form in hybrid and single composites, along with a few aggregates. The brighter domains of the filler particles in SR can be easily identified and are distributed homogenously, while there are only a few aggregates, especially in GR-filled SR composites.

As a single filler, GR was found to be highly exfoliated, with a grain size in the range of ~30 nm. However, in the hybrid form of GR + CB, the grain size increased to 81 nm [79]. This provided evidence for the restacking of graphene layers in GR owing to Van der Waals interactions with the CB and GR particles, thereby leading to an increase in the grain size of the CB + GR hybrid composite and a decline in the mechanical and electrical properties. However, the grain size of the CNT filler was ~37 nm, which was higher than that of the GR filler, and the grain size of the CB + CNT hybrid was only ~39 nm, which was lower than that of the CNT + GR hybrid [79]. Interestingly, the aggregation of CNTs in the presence of CB was lower than the aggregation observed in the CB + GR hybrid. This also shows that the dispersion of CNTs with and without CB was better than that of GR, demonstrating the superior properties and higher synergistic effect of CNTs and their hybrids with CB.

The presence of GR aggregates in single fillers and its hybrid with CB further suggests the higher roughness of their composites compared to composites based on CNTs and CNT + CB. In line with this hypothesis, it was found that the roughness of GR as a single filler was 23 nm, and that of its hybrid with CB was 22 nm [79]. However, the roughness was much lower for CNTs (18 nm) and the CNT hybrid with CB (10 nm) [79]. This further supports the uniform dispersion of CNTs and evidences their synergistic relationship with CB in the hybrid form. The better mechanical and electrical properties of the CNT + CB-hybrid-based SR composites are discussed in later sections of this review. As previously mentioned, AFM reveals the surface roughness, while phase imaging quantifies the surface stiffness, which describes the difference in the modulus of the mechanical properties of the filled composites between the two components. Recently, Lee et al. [34] employed AFM to study the filler percolation threshold for CNTs in RTV-SR composites. From the AFM results, the authors found that at 3 phr CNT, continuous long-range filler networks (or filler percolation threshold) of CNTs were observed in the RTV-SR matrix.

#### 3.2.2. Filler Dispersion through TEM

**Figure 4:**
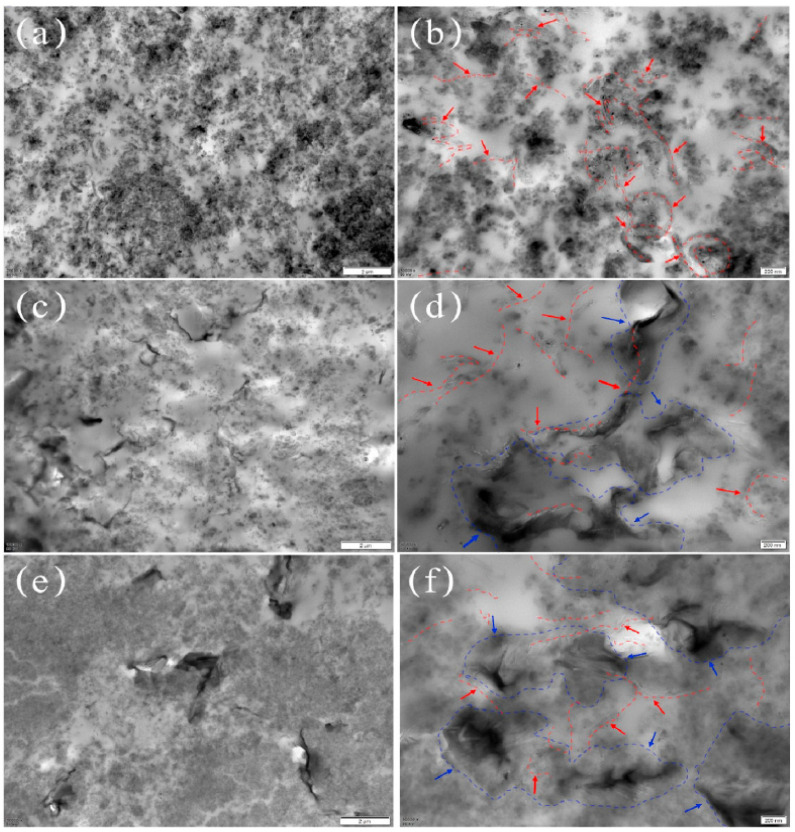
TEM micrographs of the CNT-GR/SR composites: (**a**,**b**) CNT-SR composites; (**c**,**d**) CNT-GR/SR composites; (**e**,**f**) self-assembled CNT-GR/SR composites; line indicating formation of conductive pathways in SR matrix (blue line for GR and red for CNT) [52].

TEM is frequently used to study filler dispersion in polymer matrices [80]. Figure 4 shows TEM images of the three types of carbon-based nanofillers in SR matrix, where the grey dots indicate the filler particles. The CNT particles in the SR matrix are visible in Figure 4a,b. It can be seen from the high-resolution TEM image that few CNT particles were agglomerated while continuous CNT networks were formed, as represented by the red lines [52]. In their TEM studies, Hu et al. [81] also showed the agglomeration of CNT in an SR matrix at 5 wt% CNTs. Although ultrasonic treatment was applied to the CNT before dispersion in the SR matrix, the CNTs were still not dispersed uniformly. This is due to the strong Van der Waals forces and intermolecular π–π interactions among the CNT particles [81].

It is evident that the CNT forms a filler percolation threshold owing to its high aspect ratio, which causes the CNT particles to form interconnected networks and leads to better mechanical, electrical, and thermal properties [82,83]. The inclusion of GR to form a CNT hybrid in the SR matrix is illustrated in Figure 4c,d. GR particles are found in the vicinity of the CNT particles, which gives rise to a synergistic effect on the properties of the hybrid SR composites. It was also noticed that after the addition of GR, the aggregation of CNT particles was significantly reduced, leading to improved dispersion of CNTs in the SR matrix. Moreover, the percolation phenomenon is more evident after the addition of GR to the CNT/SR composites as more GR particles are located near the CNT particles, as demonstrated by the red (CNT) and blue (GR) lines. In the case of self-assembled CNT-GR in SR composites, as shown in Figure 4e,f, the distribution of GR and CNT is narrower, and, as in the case of the hybrid filler, the CNT particles are closer to the GR particles. The percolation phenomena are indicated by the red and blue dashed lines. In addition, bridging structures were identified between the GR and CNT particles in SR composites in the self-assembled hybrid systems. This behavior enhanced the efficiency and organization of the networks. In addition to the high aspect ratio of CNTs, the close proximity of CNTs to GR particles makes them more efficient at forming filler networks and helps in attaining filler percolation at lower filler contents. Moreover, filler percolation provides a monotonic response of the electrical properties during cyclic loading of self-assembled hybrid systems.

#### 3.2.3. Filler Dispersion through SEM

SEM can be used to study the dispersion of filler particles in a polymer matrix [84,85]. Zhao et al. [86] showed that the formation of filler percolative networks involves two stages of network formation. At lower loadings (e.g., 4 wt%), there are short-range discontinuous networks. In addition, at higher filler loadings (e.g., 8 wt%), there is long-range and continuous filler-network formation. Zhao et al. [86] showed the dispersion of a GR–CB/SR hybrid at a filler loading of 4 wt%. It was found that the properties at lower percentages were worse than those at higher loadings. This accounts for the formation of short, isolated CB networks in both the space and interior arms of the GR in the SR matrix. From the SEM images shown by Zhao et al. [86], it can be concluded that CB forms continuous networks and is interconnected, as shown by the red lines. The properties at 8 wt% CB led to a very high thermal conductivity [86]. Here, the synergistic effect of the GR-CB/SR on the thermal conductivity has previously been described [86].

**Figure 5:**
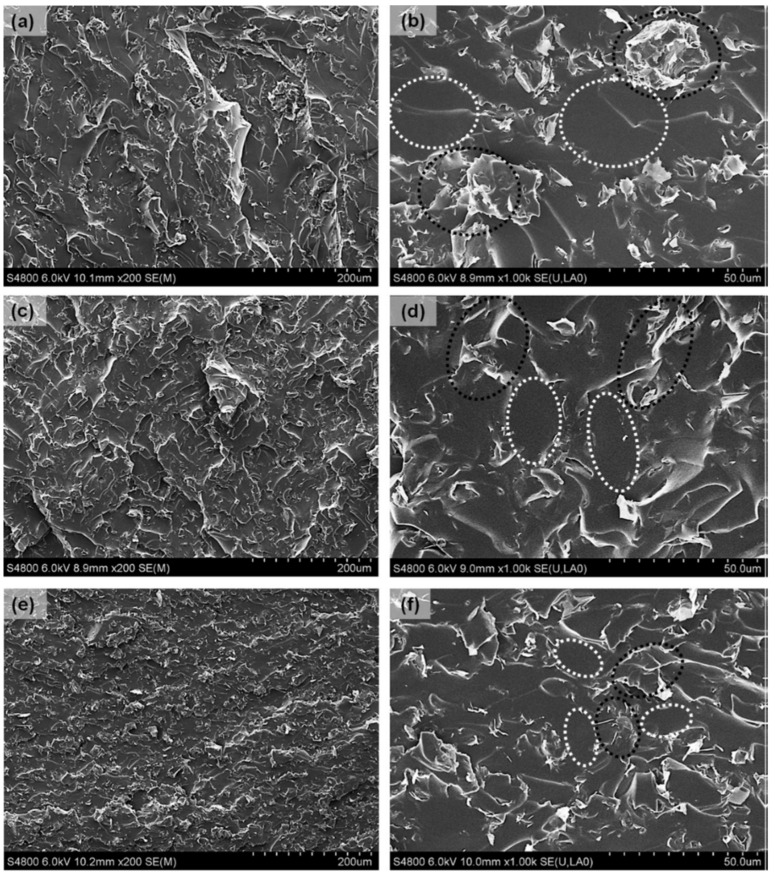
SEM images of GE/SR composites: (**a**,**b**) mechanical mixing, (**c**,**d**) solution mixing, and (**e**,**f**) ball milling. Black dotted circles denote GE-rich zones, while white dotted circles denote SR-rich zones [87].

Figure 5 further shows the microstructure and morphologies of fracture surfaces of GE/SR composites using SEM micrographs under three processing conditions: mechanical mixing, solution mixing, and ball milling [87]. The SEM micrographs revealed filler-rich zones and zones rich with matrix phases. From these investigations, they reported that the dispersion of GE sheets seems to be improved by solution mixing and ball milling. The results in Figure 5 further indicate that the size of GE agglomerates is reduced, and clusters of GE sheets become much looser than those formed during mechanical mixing due to the applied sonication and formation of conductive networks [87]. Kumar et al. [3] reported that among CB, GE, and CNTs, CNTs showed the best dispersion in RTV-SR composites. CNTs form filler percolative networks at a very low content of approximately 2 phr. Owing to their high aspect ratio (65–70), high surface area (250 m^2^/g), and improved dispersion, CNTs outperformed CB and GE in terms of the mechanical, electrical, and thermal properties of their respective composites. Thus, the study concluded that among GE, CNTs, and CB, CNTs are the best performing filler for reinforcement [3].

### 3.3. Polymer–Filler Interaction through Swelling Tests

The physical performance of a composite strongly depends on a number of factors, such as the shape, volume fraction, and particle size of the filler particle; filler–filler interactions, and especially polymer–filler interactions. The polymer–filler interaction depends on the polymer–filler compatibility [88] and the interfacial area [89]. A high interfacial area provides a greater opportunity for polymer chains to interact with the filler particles and their aggregates. The polymer–filler interaction leads to the adsorption of polymer chains on the filler surface. The interfacial interaction and dispersion of filler particles can be enhanced by introducing silane coupling agents to improve the adhesion between the filler and rubber composite phases [90].

**Figure 6:**
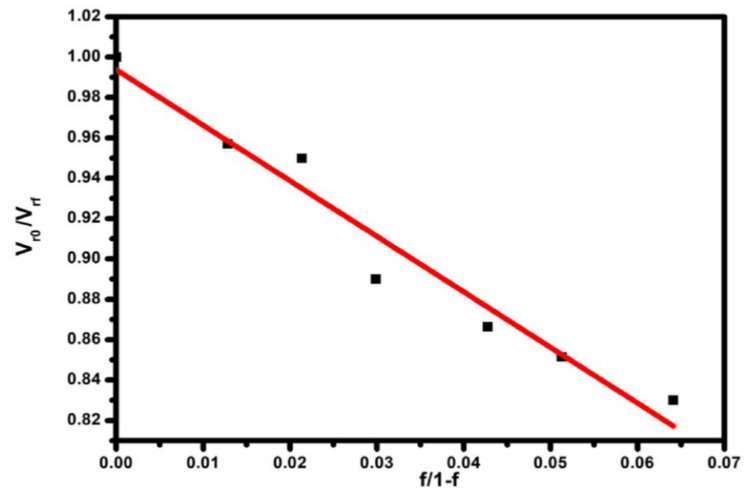
Kraus plot of EG/SR composites [95].

The mechanical properties of composites have been improved by adding stiff fillers to a soft SR matrix. An additional contribution to the reinforcement arises from the interfacial interactions between the SR and filler particles. Thus, the quality of the interfacial interaction plays an important role in determining the properties of the filled composites. These interactions lead to an increase in the degree of cross-linking, which can be determined through equilibrium swelling tests [91]. Figure 6 depicts the polymer–filler interaction using swelling methodology and a Kraus plot. Equilibrium swelling measurements are highly effective in determining the number of effective network chains in a rubber matrix. For the filler added to the SR matrix, swelling tests not only reveal the effect of chemical junctions but also the density of polymer–filler attachments.

Kraus demonstrated the theory of interaction between fillers and polymer matrix [92,93]. This theory reports the swelling of the vulcanizates in certain solvents such as toluene and was shown to obey the following equation:

Q_r_/Q_r0_ = v_r0_/v_r_ = 1 − mΦ/(1 − Φ) (2)

where Q_r_ and Q_r0_ are the individual swelling ratios of filled and unfilled vulcanizates, respectively; v_r0_ and v_r_ represent the individual volume fraction of filled and unfilled rubber composites after swelling; and m can be determined by the following equation:

m = 3c (1 − v_r0_^1/3^) + v_r0_ − 1 (3)

where c is the constant characteristic of the reinforcing filler, but it is independent of the degree of vulcanization, the nature of the solvent, and the polymer matrix. According to Kraus, if there is good polymer–filler interaction, the restriction to swollen rubber leads to a decrease in the ratio of v_r0_/v_r_ as the filler loading in the polymer composite is increased. Therefore, application of Equation (3) is useful for determining the adhesion between polymer chains and filler particles in polymer composite [94].

In this case, according to Kraus’ theory, the higher the negative slope is, the better the polymer–filler interaction and reinforcement are for the filler and the polymer matrix in polymer composites. In Figure 6, the slope is 0.95 for 3 phr expanded graphite (EG) and 0.83 for 15 phr EG in the SR matrix, indicating good reinforcement of the SR matrix by EG [95]. It can also be concluded that as the reinforcement increases, the degree of stress transfer from the polymer chains of the SR matrix to the EG particles increases, which, in turn, results in an increase in the constraint zone in the polymer chains of the SR matrix. Kumar et al. [2] also studied the strength of the interfacial interaction through a Kraus plot. The CNT specimen showed a higher slope and, thus, a higher Kraus constant than the CB-based RTV-SR composite. This is attributed to the higher interfacial interaction and high networking density of the CNT particles in the RTV-SR composite.

## 4. Properties of SR/Carbon-Nanofiller-Based Nanocomposites

### 4.1. Mechanical Properties

#### 4.1.1. Under Compressive Strain

The compressive and tensile mechanical properties depend on a number of factors, but the dispersibility and interfacial stress transfer between the filler and polymer composite are the most important [96]. It is expected that the addition of a small amount of CNTs significantly improves the mechanical properties of composites owing to the high aspect ratio and attainment of the filler percolation threshold at lower CNT amounts in the polymer composites.

**Figure 7:**
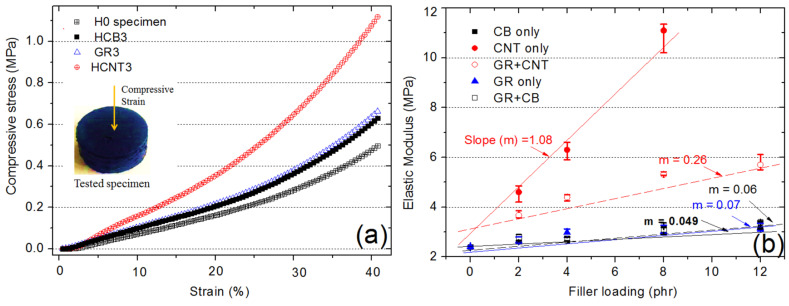
Compressive mechanical properties: (**a**) compressive stress vs. compressive strain for different fillers and (**b**) elastic modulus for different fillers [97].

The compressive mechanical properties and synergistic effects of filled vulcanizates are shown in Figure 7. Figure 7a presents a graph of the compressive stress against the compressive strain. It can be seen that the compressive stress increases with increasing compressive strain in all vulcanizates [97]. This is due to the impact of (a) the large, increasing density of the filler clusters in the polymer composites, (b) the tangling and detangling of the filler clusters at different strains, and (c) the improved interfacial adhesion and filler networking in polymer composites [98,99]. Figure 7a shows the dominant mechanical properties of CNT-based composites [97], which are attributed to (a) the orientation and de-orientation of the filler clusters, especially those with high aspect ratios such as CNTs; (b) decreasing the interparticle distance of the filler, leading to attainment of the filler percolation threshold and, hence, improved properties; and (c) a high interfacial area between the CNTs and polymer chains in the composites, which leads to efficient stress transfer from polymer chains to CNT aggregates. High stress transfer assists in efficient heat dissipation within the composite [100].

The elastic modulus was measured at a compressive strain of 0.23% and is plotted against filler loading in Figure 7b. The figure shows that hybrids based on CNTs and GR exhibit improved compressive mechanical properties (i.e., compressive stress and elastic modulus), especially at higher filler contents. This enhancement is attributed to the synergistic effects of the hybrid fillers [101] and the restriction of polymer chain mobility due to the increased fraction of bound rubber in the polymer composites. Many recent studies have shown that the improved mechanical properties of hybrid fillers in polymer composites are due to synergistic effects. The results in Figure 7 also indicate that GR and CNTs are well dispersed in the polymer matrix [97]. The improvement in compressive mechanical properties is also due to the attainment of the filler percolation threshold between CNTs and between GR and CB in the polymer composites. The high surface area of the filler provides good interfacial interaction and a high interfacial area, and thus, higher adsorption of the polymer chains on the filler particles is possible. The good interfacial interaction leads to heat dissipation from the polymer chains in the polymer composites to filler particles, resulting in improved mechanical properties [102]. Kumar et al. [2] also showed that CNTs as a filler have better mechanical properties than CB does. The authors showed that the Young’s modulus increased by 272% with 2 phr CNTs and increased to as high as 706% at 8 phr CNTs. In addition, the Young’s modulus improved by only 125% with 10 phr CB-reinforced RTV-SR composites [2]. These findings are further supported by another study in which the compressive moduli of CNT, GE, and CB were reported [3]. The authors showed that CNTs are outstanding fillers for improving the modulus and stiffness of RTV-SR-based composites [3]. They attributed the outstanding properties of CNTs to the high aspect ratio, which leads to filler percolation and long-range networks at lower filler contents (2 phr) and an exponential improvement in mechanical properties beyond the addition of 2 phr CNTs [3]. Kumar et al. [3] further showed that CNT (among CNT, CNT-GR, and GR) is the best filler for improving mechanical properties. With 3 phr CNTs, the tensile strength and Young’s modulus were 1.78 and 1.77 MPa, respectively, which are better than those of other fillers. However, the other fillers, such as GR, showed a higher fracture strain of 244% compared to CNTs with 191% [34].

#### 4.1.2. Under Tensile Strain

**Figure 8:**
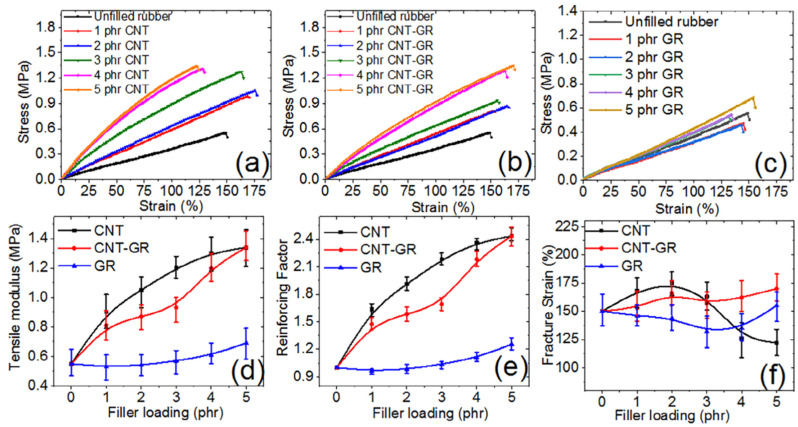
Stress–strain curves for (**a**) CNTs, (**b**) CNT-GR, and (**c**) GR. (**d**) Tensile modulus, (**e**) reinforcing factor, and (**f**) fracture strain for the studied fillers [106].

Tensile strength and fracture strain are a few of the many properties extracted through tensile mechanical measurements. The stress–strain behaviors of CNTs (Figure 8a), CNT-GR (Figure 8b), and GR (Figure 8c) are presented. From the measurements, it was found that tensile stress increased with increasing tensile strain until fracture strain. The increase in tensile stress is assumed to be due to (a) filler–filler and polymer–filler interactions, (b) efficient filler networking in the polymer composites, (c) stress transfer from polymer chains to filler aggregates, and (d) re-orientation of polymer chains and filler particles in the direction of the tensile strain. The CNT-based composites showed the highest tensile stress due to fracture strain. This is attributed to the high aspect ratio, high interfacial area, and the formation of percolative filler networks at lower CNT contents in comparison to GR [103]. The synergistic effect of the CNT and GR hybrid is shown in Figure 8b, in which the fracture strain of the hybrid species is higher than that of the individual CNT and GR filler species.

Furthermore, Song et al. [4] showed that pure SR has poor mechanical properties, such as a low tensile strength of 0.40 MPa and a fracture strain of 115.07%. With the addition of a hybrid filler at 10 phr, the tensile strength reached 4.5 MPa and the fracture strain increased to 211.15%. The mechanical properties of the SR/conductive carbon black–polymerized MPS—carbon nanotube (CCB-P-CNT) composite were significantly enhanced compared to those of the SR filled with 10 phr CNTs. This is mainly attributed to the fact that conductive carbon black (CCB) modified by polymerization of 3-trimethoxysilyl propyl methacrylate monomer (PMPS) improves the dispersibility of CNTs in the SR matrix [4]. Similarly, the addition of graphene nanoribbon (GNR) to the SR matrix improved the mechanical properties of the composites [104]. For instance, by adding 2 wt% GNR to the SR matrix, the tensile strength increased to 0.40 MPa, the fracture strain decreased to 78%, and the Young’s modulus increased to 0.85 MPa [104]. Sarath et al. [95] also showed that the addition of EG to the SR matrix improved the mechanical properties. For instance, with 7 phr EG in the SR matrix, the tensile strength increased to 6.8 MPa, the fracture strain increased to 221%, the Young’s modulus at 100% strain increased to 3.86 MPa, the tear strength increased to 30.2 Newton-millimeter (N·mm), and the hardness increased to 65 [95].

The tensile modulus (Figure 8d) and reinforcing factor (Figure 8e) were reported for different fillers and their hybrids, which increased with increasing filler content in the SR matrix. The tensile modulus and reinforcing agent were the highest for CNT-based composites. The superior properties, especially for the CNT-based composites, were due to the efficient filler networking in the SR matrix. The filler–filler and polymer–filler interactions in the SR composites were also speculated to be the reason for the improved properties upon increasing the filler content in the SR-based rubber matrix. It is also interesting to note that the tensile modulus and reinforcing factor of the CNT-GR hybrid at 5 phr were higher than those of the CNT composites. This was attributed to the synergistic effect of the CNT-GR hybrid.

The effect of fillers and their hybrids on the fracture strain is shown in Figure 8f. It can be seen that the fracture strain, especially for CNTs, increased for up to 2 phr filler content and then decreased, while for the CNT-GR hybrid and GR, the fracture strain increased steadily until 5 phr. The decrease in CNT content after 2 phr is due to the aggregation of CNT particles in the SR matrix. Moreover, the increase in fracture strain for the CNT-GR hybrid is due to the synergistic effect and lubricating effect of GR in the hybrid and as a single filler. GR flakes did not appear to significantly affect the viscosity of the composites compared to the CNT fillers. In contrast, CNTs readily form filler networks at lower filler contents because of their high aspect ratio, leading to better properties than those imparted by GR flakes. Kumar et al. [105] showed that with the use of a thinner in an SR composite, the fracture strain increases to 241% from 226% (unfilled), while the Young’s modulus drops from 0.6 (unfilled) to 0.17 MPa. Therefore, it can be concluded that the use of a thinner softens the composites and makes them useful for various applications, such as flexible devices that require softness, flexibility, and stretchability [105]. The authors further demonstrated that the dissipation losses fell sharply from almost 90 (10 phr thinner) to 22% (60 phr thinner) [105].

#### 4.1.3. Mechanical Properties: Experimental Data vs. Theoretical Prediction

The extent of reinforcement of a polymer matrix by a filler depends on various parameters of the filler, such as the shape, particle size, aspect ratio, orientation, and surface area of the filler particle, the viscosity of the polymer matrix, and its modulus. The high surface area—which is due to the small particle size of carbon nanomaterials—and especially the high aspect ratio of CNTs make them promising candidates for the reinforcement of polymer matrices. In contrast, theories and models help to project the modulus of the reinforced polymer matrix by considering the shape, size, and aspect ratio of the filler particles used in the polymer matrix. Therefore, it is interesting to compare experimental data with those predicted using theoretical models. The Guth model, which is largely based on the aspect ratio of the filler, has been widely used to predict the modulus of filled polymer composites [107,108]. The equation used in the prediction is

E’ = E’_o_ (1 + 0.67 fΦ + 1.62f^2^Φ^2^), (4)

where E’ and E’_o_ are the moduli at low deformation in filled and unfilled rubber, respectively; Φ is the filler volume fraction of the system; and f is the aspect ratio of the filler particles. The quadratic term represents the mutual disturbance among the connected filler particles. Similarly, the Halpin–Tsai model can be used to predict the modulus of filled composites [109,110]. This model largely considers the aspect ratio (f) and orientation of the filler particles. The general expression of the model is given by

E’ = E’_o_ (1 + 2 fΦη)/(1 − Φη), (5)

where η is given by

η = [(E_f_/E’_o_ − 1)/(E_f_/E’_o_ + 2f)]. (6)

**Figure 9:**
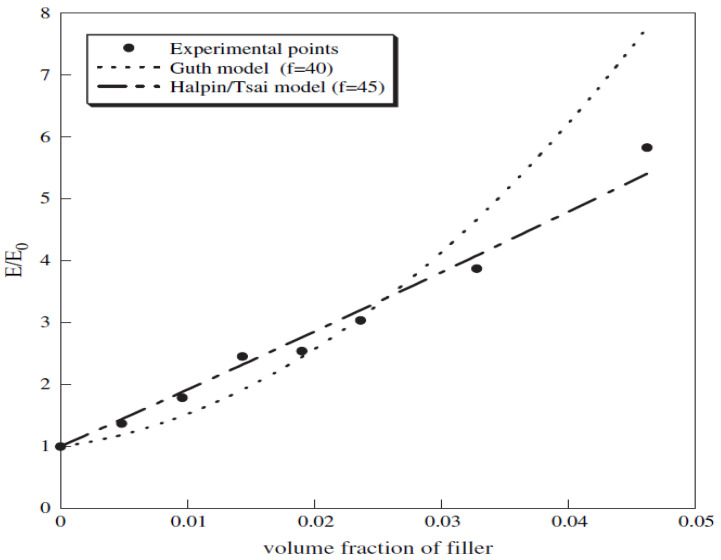
Experimental and theoretical predictions of the modulus in CNT-filled composites [111].

Based on the above models, the modulus of filled composites was predicted using the respective aspect ratio values of 40 for the Guth model and 45 for the Halpin–Tsai model and is presented in Figure 9 [111]. The behaviors of the theoretical modulus and the experimental data are depicted, and it can be seen that the experimental data fit well with the theoretical models. In addition, the Halpin–Tsai model fit more accurately than the Guth model, which deviated as the filler volume fraction increased. However, it is noteworthy that both models assumed similar aspect ratios of the filler used in the reinforcing matrix [111].

#### 4.1.4. Dynamic Mechanical Thermal Analysis

**Figure 10:**
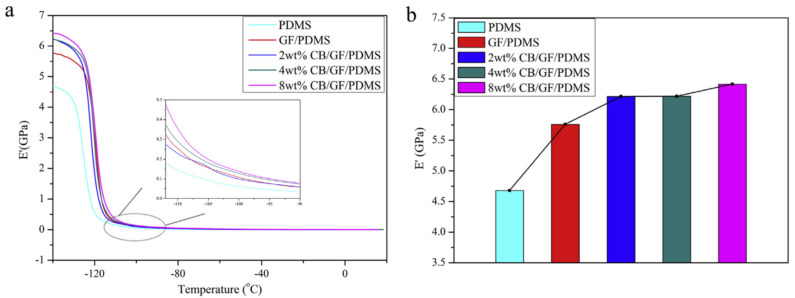
Dynamic mechanical thermal properties of CB-GF/SR composites: (**a**) storage modulus (E’); (**b**) storage modulus at −139 °C [86].

Figure 10 shows the dynamic mechanical thermal analysis of an SR matrix filled with GF and a GF-CB hybrid. Figure 10a shows that the behavior of the filled composites was identical despite the different magnitudes. In the glassy region, the addition of GF and the GF-CB hybrid causes a noticeable increase in the storage modulus, as shown in Figure 10b. In one study, incorporating 0.5 wt% GF in GF/SR composites was found to be more efficient than incorporating 2D graphene nanosheets (GNS) [86]. Eventually, by increasing the CB content from 2 to 8 wt% in the CB-GF hybrid, the storage modulus of the SR composites gradually improves. However, as the temperature increases from −139 to −110 °C, the storage modulus in Figure 10b decreases sharply as a result of glass transition phenomena [86].

Nevertheless, the rate of decrease in the modulus with increasing temperature is compensated by rigid fillers such as GF and CB, which impart thermal stability to the SR composites. Moreover, in the rubbery state, the GF-CB-hybrid-based SR composites also had a higher storage modulus than the unfilled SR and GF-filled composites (inset of Figure 10a). Despite the considerable flexibility of the polymer chains of the SR matrix under this rubbery state, the rigid GF and CB filler particles could confine the segmental motion under strain.

### 4.2. Tribological Properties

**Figure 11:**
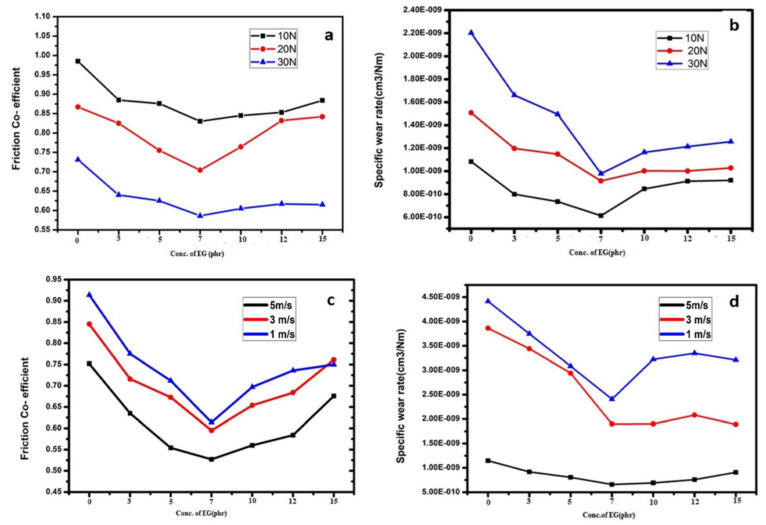
(**a**) Friction coefficients of composites; (**b**) specific wear rates at different loads; (**c**) friction coefficients; (**d**) specific wear rates at different sliding velocities [95].

Figure 11 shows the influence of the applied load and sliding velocity on the friction coefficient (COF) and wear rate. 

It is interesting to note that as the load increases, the COF decreases (Figure 11a) and the wear rate increases (Figure 11b). Moreover, the values for the EG-filled composites are lower than those of the unfilled SR composites. At a lower load, the COF was high and the specific wear rate was lower because lower loads were unable to break the EG–SR interfacial interaction in the composites. As the applied load was increased to 30 Newton (N), there was a drastic decrease in the COF and an exponential increase in the wear rate [95]. At higher loads, the interfacial interaction between EG and SR broke, which led to a decrease in the COF and an increase in the wear rate.

Figure 11c,d show the influence of the COF and wear rate on various sliding velocities. With the increase in EG content in the SR matrix, the COF and wear rate decreased. The composite with a low sliding velocity (1 m/s) exhibited a higher COF and wear rate than that with a higher sliding velocity (5 m/s). Here, it was observed that 7 phr EG was the optimum value [95]. When the composites were studied under rotation at a low sliding velocity, effective contact between the SR and counter face occurred. Therefore, the specimen with unfilled SR exhibited a higher COF and wear rate. However, with the addition of EG to the SR matrix, the lubricating properties of EG affected the COF and wear properties. In addition, EG forms lubricating films, thus reducing the contact area between the SR and EG and leading to a reduction in the COF and wear rate. However, the case with a higher sliding velocity of 5 m/s was different. Less time was needed to form EG films, if they were generated at all; if films were generated, the higher sliding velocity affected the films’ ability to remain between rubbing surfaces. In conclusion, the wear rate and COF are significantly affected by the addition of EG, the sliding velocity, and the applied load [95]. Moreover, temperature also affects the COF, wear rate, and wear mechanism.

### 4.3. Thermal Properties

#### Thermal Conductivity, TGA, and DTG

**Figure 12:**
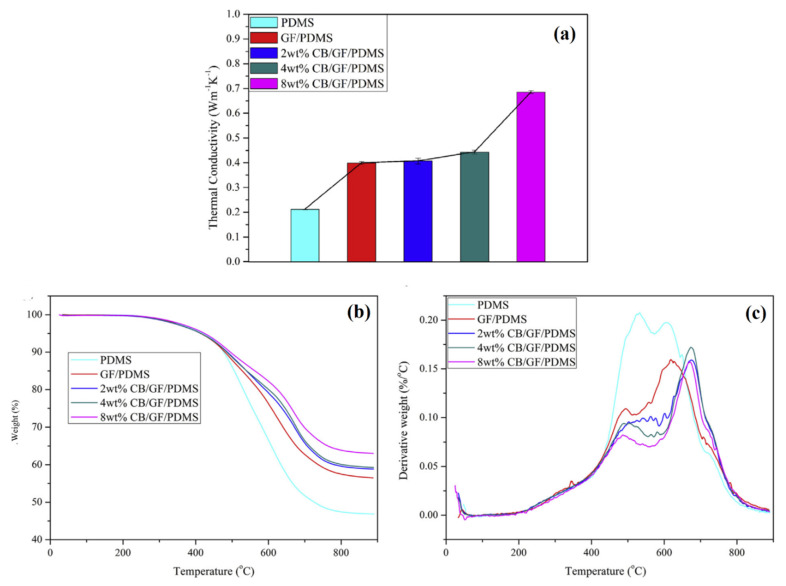
(**a**) Thermal conductivity and (**b**) TGA and (**c**) DTG curves of composites based on GF and GF-CB hybrid in SR matrix [86].

Figure 12a shows the thermal conductivities of different composites based on GF and GF-CB hybrids in SR matrix. A sharp improvement in the thermal conductivity was observed when GF was added to the SR matrix. Then, when CB was added as a hybrid filler, the thermal conductivity was almost constant up to 4 wt% of the CB-GR hybrid in the SR matrix [86]. From 4 to 8 wt%, there was a sharp increase in the thermal conductivity. This was presumed to be due to the shift from short-range and discontinuous (4 wt%) filler networks to long-range, continuous, and well-connected filler networks (8 wt%) [86]. This hypothesis was confirmed by SEM, as shown in Figure 5. In other words, at 8 wt% CB-GR hybrid, filler percolation occurs, which leads to a marked improvement in the thermal conductivity.

Hu et al. [81] also determined the thermal diffusivity of SR composites based on GF and CNTs. The thermal diffusivity of pure SR was reported as 0.105 m^2^/s, and it increased with the addition of GF and CNTs. For instance, the thermal diffusivity was 0.113 and 0.119 m^2^/s with 1 wt% GE and 5 wt% CNTs, respectively. This can be attributed to GR’s ability to strongly interact with polymer chains in the SR matrix and lower the agglomeration rate compared to CNTs [81].

Figure 12b,c show thermogravimetric analysis (TGA) and differential thermogravimetric (DTG) curves of composites based on GF- and CB-filled SR. It was noticed that weight loss was initiated as the subjected temperature increased, and it started decreasing significantly between 400 and 700 °C [86]. The weight loss was the lowest for the 8 wt% CB/GF-filled SR matrix. It is recognized to be due to the improved thermal conductivity of the composites, as described in Figure 12a. At 400 °C, the apparent weight loss of the composite was due to the breakage of Si-Si, C-H, and Si-O bonds that led to the production of volatiles such as H_2_, CH_4_, etc., and produced the first significant weight loss [86]. With the further increase in temperature from 400 to 550 °C, there was a further loss of weight, which could be due to further degradation in polymer chains producing volatile substances [86]. It is interesting to note that with the addition of CB and GF in the SR matrix, the weight loss was lower than that in virgin SR matrix as described above [86]. It is due to the increase in networking and additional polymer–filler interactions that provide higher thermal stability at higher temperature.

### 4.4. Electrical Properties

**Figure 13:**
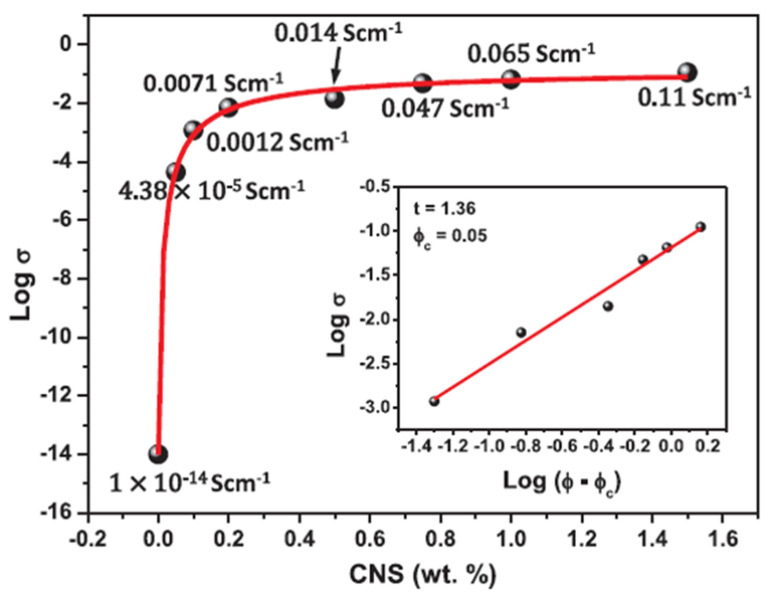
Electrical conductivity of CNS/SR composites with varying CNS contents. Inset shows logarithmic curve [53].

The electrical properties of the carbon nanostructure (CNS)-filled SR composites are shown in Figure 13. An exponential increase in electrical conductivity occurred when the CNS loading was increased from 0 (unfilled SR) to 0.05 wt%. The electrical conductivity was further increased to 0.11 S/cm with increasing CNS loading up to 1.5 wt%, which is far higher than that of the unfilled SR matrix. A drastic improvement in the electrical conductivity was observed at a particular wt% of CNS, which is known as the filler percolation threshold. The filler percolation threshold for CNS was as low as 0.05 wt%, where the electrical conductivity increased from 10^−14^ to 10^−5^ Siemens/centimeter (S/cm) [53]. The continuous and interconnected filler network of the CNS results in a low electrical percolation threshold. The aspect ratio of CNS (>3000) is also assumed to play a vital role in forming conducting networks at lower CNS contents, as demonstrated in Figure 13 [53]. The percolation threshold indicates that a minimum amount of CNS is required where CNS contact points are interconnected. A minimum average distance between the CNS particles is established to create a conductive network outside the SR matrix.

Moreover, Yang et al. [52] derived the relationship between the electrical conductivity and filler (CNT or GE) volume fraction in an SR matrix. The authors showed that as the GE and CNT contents in the SR matrix increased, the electrical conductivity increased exponentially. The results revealed a synergistic effect in the CNT-GE hybrid, which demonstrated higher electrical conductivity than that of GE and CNT as single components [52]. The percolative thresholds were 1.97 wt% (CNT), 1.27 wt% (CNT-GE hybrid), and 0.92 wt% (self-assembled CNT-GE hybrid) composites [52]. These values are slightly higher than those reported for the CNS/SR composites in Figure 13 [53]. Song et al. [4] determined that the percolation threshold for the SR/CCB-P-CNT composite is 0.30 phr. However, this value is lower than that reported by Yang et al. [52] and higher than those reported in Figure 13 [53]. In addition, the electrical conductivity of the composite reported by Song et al. [4] was found to be affected by vulcanization by the SR matrix. This is attributed to the shrinkage between SR molecular chains, which leads to the destruction of the conductive network formed in the SR matrix and a decrease in electrical conductivity [4]. After vulcanization, the electrical percolation shifts from 0.39 to 0.55 phr [4]. In another study by Hu et al. [81], electrical measurements showed that the volume resistivity decreased with the addition of GF and CNTs. In this study, the percolation threshold was approximately 2 wt% for graphene and 5 wt% for CNTs. These values are quite high compared to those reported by Song et al. [4], Yang et al. [52], and in Figure 13 [53]. The lower values of the percolative networks for GF than for CNTs reported by Hu et al. [81] can be attributed to the uniform distribution of GF particles compared to the aggregated CNT particles in the SR matrix. These aggregated CNTs lead to a lower tendency to form long-range percolative networks, and thus, a higher amount of CNTs is required. Lee et al. [34] studied resistance as a function of tensile strain for CNT and CNT-GR hybrid fillers. The authors found that the resistance at fracture strain for 3 phr filler was 0.4 kΩ for CNTs, which is much lower than that for the CNT-GR hybrid (59.4 kΩ) [34].

## 5. Applications

### 5.1. Piezoresistive Response of CNS/SR Composites under Cyclic Strain

**Figure 14:**
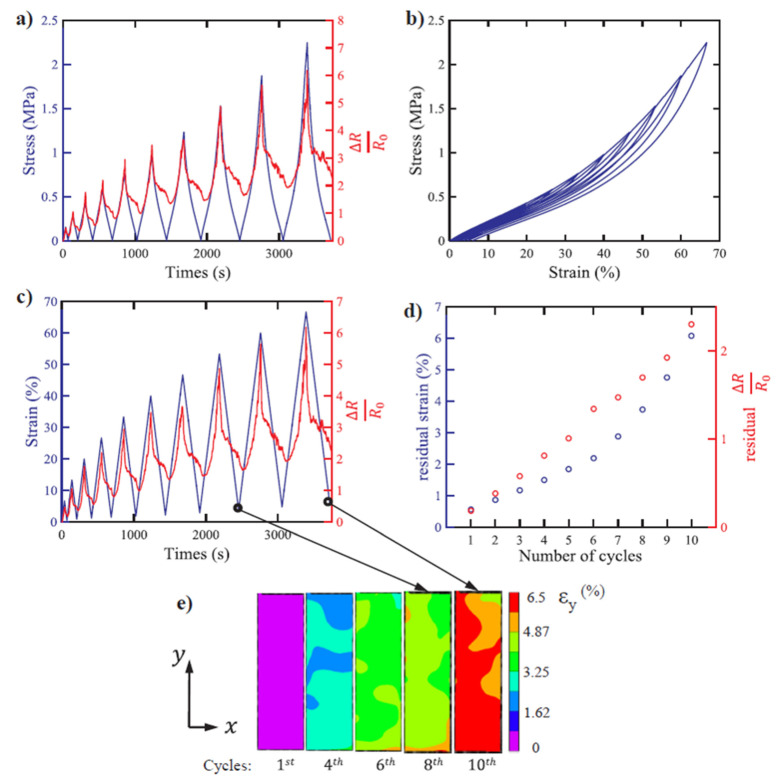
Cyclic strain and relative resistance with increasing amplitude: (**a**) stress and relative resistance; (**b**) multi-hysteresis of stress–strain curve; (**c**) strain and relative resistance; (**d**) number of cycles against residual strain and residual relative resistance; (**e**) contour map of residual strain at different cycle numbers [53].

Figure 14a–d show the piezoresistive response and durability of specimens under cyclic strain with increasing amplitude. The relative resistance can be correlated with the applied stress–strain cycles (stretch–release cycle), as the relative resistance increases under stretching and decreases as the stress is released. Under cyclic strain, the system generated a residual strain in each subsequent loading cycle. This residual strain and accumulation of resistance can be attributed to the viscous nature of the SR matrix. Hence, in addition to the self-sensing capability of these systems, the residual strain and residual resistance can be considered. Arif et al. [53] also showed that when the strain is zero in the unloading cycle during the cyclic process, the resistance does not return to its initial position and leads to residual resistance and residual strain in the SR composite. This is due to the permanent destruction of a few interactions in the conductive pathways made by filler particles in the SR matrix under multi-hysteresis cyclic loadings [53]. The destruction to the permanent conductive networks of the SR composites can be obtained by measuring the volume resistivity based on the correlation between the resistivity and inter-particle distances of the conductive filler particles [53].

Figure 14e shows a strain contour map of the residual strain obtained after the release of different loading cycles. The distribution of strain can be observed at the macroscopic level in the contour map. Using the residual strain and residual resistance, one can predict the zones of damage and failure initiation. For example, macroscopic strain localization could indicate higher damage because of the local CNS network arrangement [53].

### 5.2. Strain Sensors

**Figure 15:**
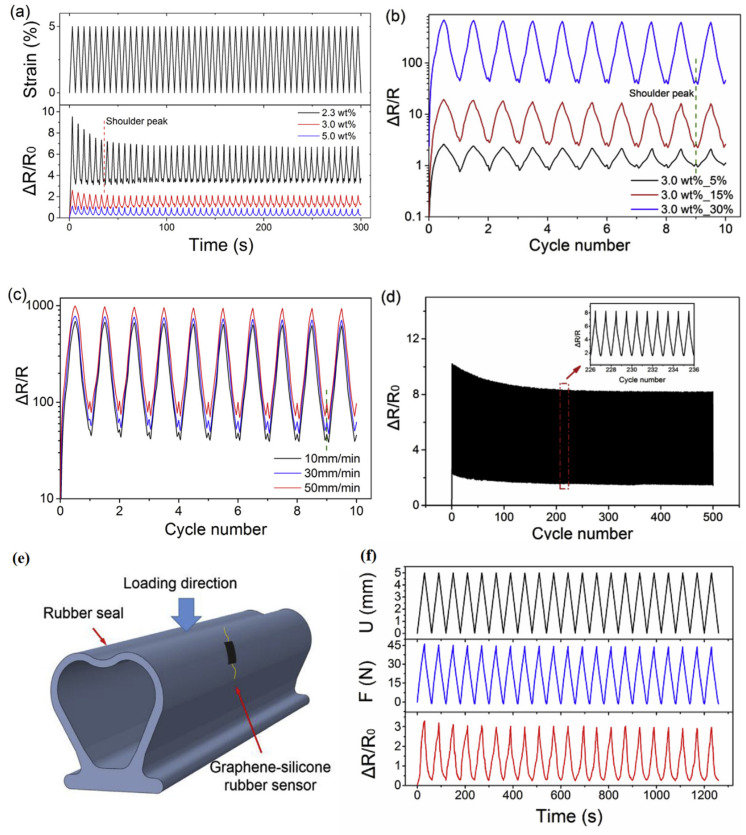
Change in resistance upon applied cyclic strain: (**a**) change in resistance at increasing GE content in SR matrix; (**b**) change in resistance at increasing strain from 5 to 30%; (**c**) change in resistance at different strain rates increasing from 10 to 50 mm/min; (**d**) durability cycle for the composites at 30% strain and strain rate of 10 mm/min; (**e**) application of the strain sensor for monitoring on a rubber seal; (**f**) cyclic response of the strain sensor under different cycling strains [1].

The stretchability and strain sensitivity of GE/SR composites have been recorded. Thus, cyclic strain was applied and various strain-sensitive measurements, such as changes in resistance upon cyclic strain and durability, were measured [1]. Figure 15a shows the response of the change in resistance as a function of increasing GE content in the SR matrix. The change in resistance increased gradually with increasing strain, but the process of change was non-monotonic. The “shoulder peak” phenomenon was observed for these GE-based SR composites. Moreover, it was found that the second peak increased gradually with increasing cyclic loading and stabilized after a few cycles. The deconstruction and reconstruction of conductive GE networks, which stabilized after a few cycles, were believed to cause such a response. Figure 15b shows the change in resistance as a function of increasing strain from 5 to 30%. It can be noted that the resistance change was higher at higher strain rates (30%), which was assumed to be due to the higher discontinuity and increasing interparticle distance in conductive GR networks at higher strain rates [1]. In addition to the strain magnitude, the strain rate also affects strain-sensitive properties. Yang et al. [1] studied the relative resistance as a function of cyclic strain for CNTs, a CNT-GE hybrid, and a self-assembled CNT-GE hybrid. The authors found that the relative resistance was higher for the first cycle and then stabilized in subsequent cycles, which is related to the viscoelasticity of the SR matrix. Similar behavior was reported by the authors in Figure 15a,b.

Figure 15c shows the change in resistance as a function of the strain rate increase from 10 to 50 mm/min. The results show that with an increase in the strain rate, the change in resistance increases. The results also highlight that a change in the resistance at higher strain rates causes higher strain sensitivity. This could be due to the higher stress applied to the materials induced by the higher strain rate. Another reason could be that the destruction of larger GE networks at higher strain rates led to a higher change in resistance. Figure 15d shows the durability cycle of the GE/SR composites over 500 cycles. It was found that as the number of cycles increased, the change in resistance initially decreased and then stabilized. This can be attributed to the fresh breakdown and reformation of the GE networks, which stabilized after certain cycles. These features indicate that the GE/SR composites have good stability and durability. In addition, based on these measurements, the GE/SR composites possess good fatigue properties and a higher ability to restore stress. Kumar et al. [3,106] also studied the durability of strain sensors for CNT-, CNT-GR-, and CB-based composites. From the results, they found that the resistance loss was negligible, indicating high electromechanical stability for the CNT-, CNT-GR-, and CB-based composites used as strain sensors [3,106].

Figure 15e shows the use of a strain sensor based on a GR/SR composite firmly bound by a rubber seal for monitoring. Both compressive displacement and compressive load are important factors in monitoring the rubber seal. The strain sensor based on the GE/SR composite could monitor both these states and generate electrical signals with different compressive loads. Figure 15f shows that the relative resistance of the strain sensor based on the GR/SR composite changed gradually when the rubber seal underwent compressive displacement. Generally, the relative resistance increases when the rubber seal undergoes compressive displacement and decreases when the rubber seal recovers [1]. Moreover, the resistance of the flexible strain sensor was very stable under strain cycling, indicating that the GE/SR composite sensor can be successfully used in sensing strain on a rubber seal.

Deformation recovery is an important performance indicator for strain sensors. The deformation recovery rate demonstrated by Song et al. [4] showed that the composites based on the SR matrix possessed a high recovery rate. For instance, the deformation recovery rate was 99.8% at 20% strain, which decreased to 97.1% with a strain from 50 to 200%. These results indicate that the SR/CCB-P-CNT composite exhibits good deformation recovery performance as a strain sensor [4]. Moreover, Song et al. [4] reported an SR/CCB-P-CNT composite with excellent mechanical properties, high strain sensitivity, and good electrical properties that could be used to detect human motions. The authors showed a change in resistance while bending and releasing a finger during drinking, demonstrating a highly sensitive strain sensing application [4].

For strain sensors, the gauge factor, which is defined as the change in the relative resistance against the applied strain, is important in addressing their application. Yang et al. [1] estimated the gauge factor for GR/SR composites at different strains. The gauge factors were 143 for 2.3 wt%, 76.7 for 3 wt%, 50.7 for 4 wt%, and 30.3 for 5 wt%. It is interesting to note that the strain sensing range increased from 35 to 170% as the GE content increased from 2.3 to 5 wt% [1]. Kumar et al. [3,106] studied the gauge factors for strain sensors based on CNTs, a CNT-GR hybrid, and CB. The authors found that the RTV-SR strain sensors based on CB and the CNT-GR hybrid had higher gauge factors of 7.8 and 32.4, respectively, compared to that for CNTs (1.3) [3,106]. The authors further studied the transient response to applied strain for CNT- and CB-based composites. From the measurements, they found that CB- and CNT-GR-hybrid-based strain sensors yielded better responses than those from CNT-based composites in strain sensors [3,106]. These results are consistent with those obtained for the gauge factor. Moreover, both composites showed a decrease in resistance during the strain-holding period. This is attributed to the stress relaxation of the rubber matrix [3].

### 5.3. Electromagnetic Interference Shielding and Microwave Reflectivity

**Figure 16:**
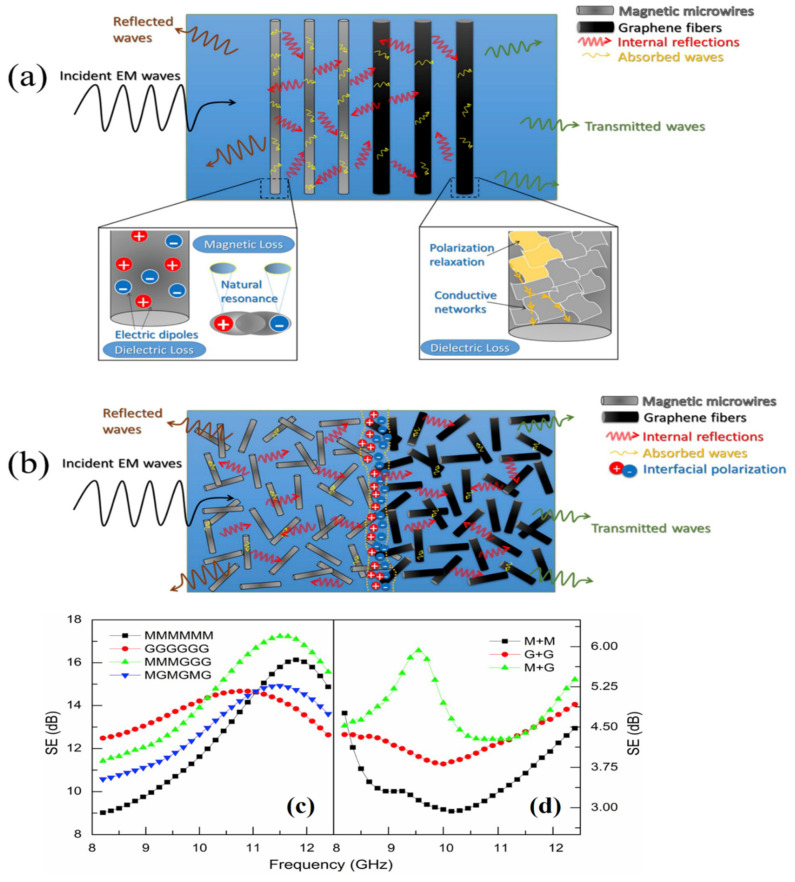
Mechanism of electromagnetic wave propagation from the specimen investigated: (**a**) periodic distributed microwave/graphene fibers; (**b**) randomly distributed microwave/graphene fibers; (**c**) shielding effectiveness of composites for periodic arrays; (**d**) randomly dispersed microwave or graphene fibers and their combinations [112].

Figure 16a,b show the mechanism behind the shielding effectiveness for periodic and randomly distributed microwave/GE fibers. It is well known that when an electromagnetic wave penetrates the specimen under investigation, the wave exhibits several reflections depending on the structure it encounters on the way. As the wave interacts several times with the structure, it subsequently loses energy [112]. The incident microwave energy exhibited two loss phenomena. The first phenomenon was related to the energy absorption of the electrical components of the electromagnetic field, and the second phenomenon was related to the absorption of the ferromagnetic resonance. The other magnetic losses could be due to the magnetic hysteresis property of the wire.

The shielding component of the graphene fibers is mainly due to dielectric losses. The formation of conductive pathways by graphene fibers results in reduced relaxation times and improved dielectric losses [112]. In addition, the defects and residual oxygen-containing functionalities in the graphene fibers also improve the polarization losses. Figure 16c,d show the shielding effectiveness of periodic arrays and randomly dispersed microwave or graphene fibers and their combinations. In the MMMGGG array with the highest SE, most of the waves were absorbed by both the magnetic and dielectric properties of the subsequently arranged microwaves. The remaining waves could be partly absorbed by the GGG fillers or transmitted through them (Figure 16a). In this arrangement, the microwaves gathered to form a thick absorbing layer, and the graphene fibers of the next layer of the composite behaved as a microwave-reflecting layer. Such a layer arrangement yields an “absorb–reflect–absorb” mechanism. However, in the case of MGMGMG arrays, the absorption of the incident wave as it propagates is less efficient due to the presence of graphene fibers between the microwires, which absorb only the electric part of the electromagnetic wave. The aspect ratio of graphene fibers plays an important role in the SE of the composites. A larger aspect ratio will greatly decrease the demagnetizing field and increase the field efficiency and loss due to absorption. A detailed discussion of the mechanism of electromagnetic wave propagation and SE phenomena is described by Xu et al. [112].

## 6. Conclusions

The present review highlights that the use of carbon-based nanofillers such as CNTs, GR, and CB in SR-based composites can significantly improve their mechanical, electrical, thermal, and tribological properties. These improved properties have significance for various applications, such as strain sensors. CNT-based SR composites exhibit outstanding properties, and CNTs are the best fillers for different applications. This review summarizes the most recent advancements of carbon-based nanofillers in the SR matrix for various applications, bridging the gaps in the existing literature in terms of the different properties and applications reviewed. It is clear from our analysis that CNTs are outstanding nanofillers among the various carbon-based nanofillers, especially in the SR matrix. Target applications such as strain sensors were found to depend on the electrical conductivity, mechanical stiffness, and stretchability of the SR-based composites. The superior properties of CNTs are due to their favorable morphology, high aspect ratio, and high surface area.

### Current Trend and Future Perspectives

The current demand for carbon-based nanofillers surged in the last 1–2 decades, especially in rubber composites such as silicone rubber. This increase in demand is due to their outstanding mechanical, electrical, thermal, and tribological properties. In the last century, carbon black was traditionally used as a filler, especially in tires, until the discovery of carbon nanotubes in 1991 by Iijima [113,114]. Over the last decade, a trend of increasing publications on CNT-based polymer composites has been witnessed. This increase in interest for CNT-based polymer or rubber composites is due to achievement of outstanding physico-chemical properties at very low loadings of below 3 phr. CNTs were extensively used as fillers until graphene was synthesized by Geim et al. [115]. The major limitation of using CNTs in rubber composites is the poor barrier properties of CNT-based composites. However, graphene has been quite promising in improving its barrier properties due to its sheet-like morphology. In addition to CNTs and graphene, nano carbon black has been used over traditional carbon black. The advantage of using nano carbon black is in its ability to provide optimum and desired properties at a lower content in polymer composites (below 20 phr) than traditional carbon black filler (around 60 phr). Thus, the difficulties faced in reinforcing with traditional carbon black, such as altered viscoelastic properties, can be overcome by using nano carbon black.

The future perspective for rubber composites based on silicone rubber and carbon nanofillers is bright and promising. Without hesitation, researchers are expected to continue searching for new possibilities of using these new materials until any further advancement or new discovery is achieved. However, challenges such as the bulk synthesis of graphene are still a matter of concern in using them at a large scale. To some extent, CNTs have achieved large-scale production and availability. In the same way, graphene forms such as multi-layer graphene and graphene nanoplatelets continue to be useful as alternatives until monolayer graphene synthesis is achieved at a large scale [116]. These graphene forms are still promising as new materials to be used as nanofillers for rubber composites. Silicone rubber as a host matrix for rubber composites has been promising for various applications, such as strain sensors. There are a number of advantages of using silicone rubber, such as its softness, easy processing, high aging and thermal resistance, high durability, and good flexibility, as desired for strain sensors. However, silicone rubber has poor fracture strain, but this limitation is not significant, and it is still the material of choice for flexible devices such as strain sensors.

## Data Availability

Not Applicable.

## Abbreviations and Symbols

CB = carbon black; phr = per hundred parts of rubber; CNT = carbon nanotube; GE or GF = graphene; MWCNT = multi-walled CNT; nm = nanometer; SR = silicone rubber; RTV-SR = room-temperature-vulcanized SR; AFM = atomic force microscopy; TEM = transmission electron microscopy; SEM = scanning electron microscopy; MLG = multilayer graphene; RPA = Rubber Process Analyzer; nano-CB = nano carbon black; GR = nanographite; FLG = few-layer graphene; GNP = graphene nanoplatelet; EG = expanded graphite; GNF = graphite nanoflake; XRD = X-ray diffraction; λ = wavelength; Å = Angstrom; β_hkℓ_ = width at half height; θ_hkℓ_ = diffraction angle; FTIR = Fourier-transform infrared spectroscopy; XPS = X-ray photoelectron spectroscopy; GO = graphene oxide; eV = electron volt; O/C = oxygen to carbon ratio; π–π = pi–pi; Q_r_/Q_r0_ = individual swelling ratio of filled and unfilled vulcanizates; v_r0_/v_r_ = individual volume fraction of filled and unfilled rubber composites after swelling; EG = expanded graphite; CCB-P-CNT = conductive carbon black–polymerized MPS—carbon nanotube; CCB = conductive carbon black; PMPS = polymerization of 3-trimethoxysilyl propyl methacrylate monomer; GNR = graphene nanoribbon; N-mm = Newton-millimeter; E’ and E’_0_ = moduli in the filled and unfilled rubber; Φ = filler volume fraction; f = aspect ratio of filler; η = viscosity; GNS = graphene nanosheet; COF = coefficient of friction; N = Newton; CNS = carbon nanostructures; TGA = thermogravimetric analysis; DTG = differential thermogravimetric curve; S/cm = Siemens/centimeter.
